# Effect of additives and filling methods on whole plant corn silage quality, fermentation characteristics and *in situ* digestibility

**DOI:** 10.5713/ab.20.0804

**Published:** 2021-03-10

**Authors:** Ting Jiao, Zhaomin Lei, Jianping Wu, Fei Li, David P. Casper, Jianfu Wang, Jianxin Jiao

**Affiliations:** 1 College of Grassland Science, Key Laboratory of Grassland Ecosystem, Gansu Agricultural University, Lanzhou 730070, China; 2 College of Animal Science and Technology, Gansu Agricultural University, Lanzhou 730070, China; 3 Animal Husbandry, Pasture and Green Agriculture Institute, Gansu Academy of Agricultural Sciences, Lanzhou 730070, China; 4 Casper’s Calf Ranch, 4890 West Lily Creek Road, Freeport, IL 61032, USA

**Keywords:** Corn Silage, Filling Times, Silage Additives, Silage Quality

## Abstract

**Objective:**

This project aimed to evaluate the effects of both different additives and filling methods on nutritive quality, fermentation profile, and in situ digestibility of whole plant corn silage.

**Methods:**

Whole plant corn forage harvested at 26.72% dry matter (DM) was chopped and treated with two filling methods, i) fill silos at one time (F1), ii) fill silos at three times (F3), packing samples into one/three silo capacity at the first day, another one/three capacity at the second day, then one/three at the third day, three replicates. For each replicate, samples were treated with three additives, i) control (CTRL, no additive), ii) Sila-Max (MAX, Ralco Nutrition Inc., Marshall, MN, USA), and iii) Sila-Mix (MIX, Ralco Nutrition Inc., USA). With three replicates of each secondary treatment, there were nine silos, 54 silos in total. Each silo had a packing density of 137.61 kg of DM/m^3^. All silos were weighed and stored in lab at ambient temperature.

**Results:**

After 60 d of ensiling, all items showed good silage fermentation under MAX filled one time or three times (p<0.01). Higher silage quality for all additives was obtained at filling one time than that filled three times (p<0.01). The highest DM and lowest DM loss rate (DMLR) occurred to MAX treatment at two filling methods (p<0.01); Digestibility of acid detergent fiber, neutral detergent fiber (NDF), and curde protein had the same results as silage quality (p<0.01). Yield of digestible DM and digestible NDF also showed higher value under MAX especially for filling one time (p<0.05).

**Conclusion:**

All corn silages showed good fermentation attributes (pH<4.0). The forage filled one time had higher silage quality than that filled three times (p<0.01). MAX with homofermentative lactic acid bacteria enhanced the lactic acid fermentation, silage quality and nutrient digestibility, and so improved the digestible nutrient yield.

## INTRODUCTION

China is an agricultural country with abundant straw resources. More than 8.4 billion tons of straw is produced every year [1]. In traditional agriculture, straw is mainly used for fertilizer, fuel and feed without any treatment [2]. Only a small part of straw is treated as feed [3]. Low protein, poor palatability and low digestibility restrict the use of straw for ruminants, leading to a waste of natural resources. Therefore, how to improve the utilization rate of straw feed is really essential. Ensiling is one of the most widely practiced processing methods. Corn silage forms the bulk of most dairy cattle rations in China and is a process that preserves valuable feedstuff for a long period of time [4]. Different fermentation aids and processing methods affect silage fermentation characteristics and quality. Silage additives affect fermentation patterns and aerobic stability in different ways according to their specific mode of action [5]. These additives are added to prevent or reduce the growth of undesirable microorganisms in silage and thus enhance silage fermentation and aerobic stability [6]. One of these additives, organic acid with strong antifungal properties has been used to increase silage aerobic stability [7]. And another additive such as acetic acid bacteria was used to improve silage aerobic stability after discovering high concentrations of acetic acid in aerobically exposed corn silages contaminated with high populations of *Acetobacter pasteurianus* [8]. And, interestingly, there was a contradictory notion that buffered acid mixtures stimulated ethanol [9]. For which notion there is an affirmative need to verify in practice. And more, ensiling is impossible to complete in one day if the silage volume is large, often requiring several days to complete. However, differences in the changes that feed quality undergoes during ensilage between these two situations are still not known. Therefore, the objective of the experiments was to evaluate the effects of various existing chemical additives, filling types and their interactions by analyzing corn silage fermentation characteristics, nutritional value, nutrient digestibility, and the yield of digestible nutrients in corn silage.

## MATERIALS AND METHODS

### Forage and treatments

Whole plant corn forage (unknown variety, approximately 26.72% dry matter [DM]) at dough stage maturity, harvested by a forage chopper (680, Juancheng Mechanical Equipment Co., Guangzhou, China) on October 1, 2015, on a dairy farm in Lintao county, Gansu, China was chopped to a theoretical length of cut of 1 cm [10]. Samples were collected to determine initial DM and nutrient composition. The chopped sample was manually packed into laboratory silos (polyethylene bottles with screw caps, 20 L capacity). There were two filling methods, i) fill silos at one time (F1), ii) fill silos at three times (F3), that is packing samples into one/three capacity of the silo at the first day, another one/three capacity at the second day, then the last one/three at the third day, three replicates of each treatment. For each replicate, the sample was treated with three additives, i) control (CTRL, no additive), ii) Sila-Max (MAX, Ralco Nutrition Inc., Marshall, MN, USA), and iii) Sila-Mix (MIX, Ralco Nutrition Inc., USA). Max contained dried *L. plantarum*, dried *E. faecium*, *P. acidilacitici*, dried *P. acidipropionici*, fruct-oligosaccharide and starch, with adding dosage of 2.5 g/t fresh forage, dissolved in 4 L deionized water, then sprayed on forage evenly, supplied a final application rate of 2.5×10^8^ of lactic acid bacteria (LAB)/kg fresh forage; Mix contained the following active ingredients: lactic acid (LA), dried *L. plantarum*, dried *P. acidilactici*, dried *E. faecium*, dried *P. acidipropionici*, dried *Bacillus subtilis*, dried *Aspergillus*, fructo-oligosaccharide, starch, iron oxide and cobalt-lactic, with adding dosage of 1.0 kg/t fresh forage, the same method on forage, supplied a final application rate of 1.8×10^6^ of LAB/kg fresh forage. The same amount of deionized water was also applied to CTRL. With three replicates of each secondary treatment, there were nine silos, 54 silos in total. Each silo had a packing density of 137.61 kg of DM/m^3^. All silos were weighed and stored in lab at ambient temperature. After 60 d of storage, the silos were weighed, opened, and samples were collected for measurement of nutrients concentrations, nutrients digestibility, pH, ammonia, volatile fatty acid (VFA), LA, and lowest DM loss rate (DMLR).

### Chemical analyses

Both fresh juice and silage juice were extracted by blending 10 g forage (wet basis) in 90 mL of distilled water through double-layered cheesecloth and filter paper (Xinhua Co, Hangzhou, China). The mixture was stored for 24 h at 4°C in a refrigerator [11], and the filtrate was used for pH, ammonia-N, LA, and VFA determination. The pH was directly measured using a pH meter (PHS-3C, Youke Instrument Co., Shanghai, China); NH_3_-N was measured using phenol-hypochlorite method according to Broderick and Kang [12]. LA and VFA concentrations were determined with a high-performance liquid chromatography (HPLC) system using an SB-AQ C_18_ column and G132B ultraviolet fluorescence detector (1260 Infinity, Agilent Technologies, Boston, MA, USA). The total VFA concentrations include LA concentrations.

Both fresh and silage samples were dried by placing them in a forced-air oven at 65°C for 48 h to determine DM and then were ground to pass through a 1 mm screen using a grinding mill (SJP-500 A Jinsui Mechanical Equipment Co., Yongkong, Zhejiang, China) and stored for analysis [13]. The crude ash (Ash) concentration was measured by placing a 1 g sample in a muffle furnace set at 560°C for 5 h. Total nitrogen (TN) was measured using the Kjeldahl method, and crude protein (CP) was calculated as N×6.25. Ether extract (EE) was determined through ether extraction. Crude fiber (CF) was measured using acid and alkali treatment. Nitrogen free extract (NFE) was calculated as 100%–H_2_O%–CP%–CF%–Ash%–EE%. The neutral detergent fiber (NDF) and acid detergent fiber (ADF) were determined according to the methods of AOAC [13]. Hemicellulose (HC) was calculated according to the formula HC = NDF–ADF.

All the chemical analyses were conducted in triplicate, and the results were expressed on a DM basis except for DM content (% fresh matter) and NH_3_-N (% TN).

### *In situ* nutrient digestibility

Six Dorset×Small Tail Sheep (Local Breed) rams with ruminal cannula and an average of 59.2±6.3 kg (mean±standard deviation) body weight were used to measure nutrients ruminal digestion. All animals were cared for according to the Chinese standards for the use and care of research animals ([2020] NO.10, GAU). The total mixed ration (TMR) was formulated to meet the nutrient requirements of a ram gaining 50 g/d according to the mutton sheep breeding standards (NY / T816-2004), Agricultural Industry Standards of the People’s Republic of China. The forage to concentrate ratio was approximately 70:30 (1.6 kg/d corn straw silage and concentrate supplement). The ingredient and nutrient compositions of the TMR are given in Table 1. The TMR was mixed daily (Animal Husbandry Machinery Co., LTD., Hebei, China) and provided twice daily at 9:00 A.M. and 5:00 P.M. with *ad libitum* access to drinking water.

The DM, NDF, HC, and ADF disappearance were mea sured according to the standardized procedures of Harazim and Paveleck [14]. Five grams of sample were weighed and sealed in nylon bag (4 cm×5 cm) with 38 μm pore size (Gansu Alvi Scientific Instrument Co. LTD, Lanzhou, China). Bags were placed into the rumens of the six sheep and incubated for 48 h, totaling six bags for each treatment. Upon removal from the rumen, bags were rinsed immediately under cold tap-water with subsequent washing in a tub with 38°C water until the rinse water was clear. The residues were dried, weighed and ground to pass through a 1 mm screen using a grinding mill (SJP-500A Jinsui Mechanical Equipment Co., China). Then, the samples were thoroughly mixed and analyzed for nutrient composition as previously described.

### Nutrient yields

Yield of DM (g/kg) was calculated according to DM content, yield of NDF (g/kg) was determined by NDF concentration. Yield of digestible DM (dDM, k/kg) and digestible NDF (dNDF, g/kg) were calculated according to DM and NDF yields and their digestibility, respectively.

### Statistical analyses

All data were subjected to least-squares analysis of variance using the PROC general linear model procedure of SAS (SAS Institute Inc., Cary, NC, USA) [15]. The model included the main effect of three additives (ADD) and two filling methods (FMs) and their interaction. Duncan’s multiple range tests were used to detect statistical significance between treatment groups. In all cases, p≤0.05 was considered to be statistically significant.

## RESULTS

### Effects on silage fermentation characteristics and nutrient composition

Significant differences were found on corn silage fermentation characteristics between different treatments at two filling times. All items showed good silage fermentation results under MAX treatment at either filling one time or three times (p<0.01). With respect to filling time, there was higher silage quality under all additives treated with filling one time than those treated with three times (p<0.01). There were significant differences for all fermentation indexes under interactions of FM×ADD (p<0.01) except propionic acid. Although the pH was similar among treatments under different filling methods, it was lower under MAX and MIX than that under CTRL for F1 (3.48 and 3.58 vs 3.80 on average, respectively), but there was the same value of 3.23 under different ADD treated with F3. The content of LA treated with MAX improved by 24.09% and 0.62% than CTRL and MIX at F1 while improved by 24.19% and 4.39% at F3 respectively. For acetic acid, propionic acid and butyrate acid, all of them treated with MAX and MIX were lower than those treated with CTRL either at F1 or F3. And the value of acetic acid/propionic acid treated with MAX was significantly higher than that of MIX and CTRL at two FMs (Table 2). Except for DM, DMLR, and EE, the other nutrients in corn silage were not affected by either FM or ADD. The highest DM content and lowest DMLR occurred in MAX treatment either at F1 or F3 (p<0.01). In fact, the nutrients such as CP, NFE, ADF, and HC showed a higher forage quality under MAX and MIX treatments compared to CTRL, although there were no significant differences among them (p>0.05) (Table 3). But compared to the initial nutrients before ensiling, the content of DM, CP, NFE, NDF, ADF, and HC decreased after 60 d ensiling, except for CF, EE, and Ash.

### Effects on digestibility and yield of digestible nutrients of corn silage

The ADF digestibility (ADFD), NDF digestibility (NDFD), and CP digestibility (CPD) of silage treated with MAX at two filling times were higher than those treated with CTRL and MIX, but they were not different between treatments except for CPD. For each treatment, filling times affected the results significantly (p<0.01); ADFD, NDFD, and CPD of each additive filled one time were all higher than those filled three times (p<0.01). The interaction between FM and ADD showed no differences (Table 4). The yield of dDM and dNDF at two FMs showed higher results under MAX treatment. For same ADD, dDM and dNDF of silage forage filled one time were higher than those filled three times (p<0.01) (Table 4).

## DISCUSSION

### Fermentation profile, silage quality and yields of digestible nutrients

After 60 d of ensiling, all corn silage showed excellent fermentation characteristics, which was indicated by dominant LA content as well as negligible propionic and butyric acid. This change can be ascribed to the presence of a sufficient fermentable substrate of the whole corn plant in the milk stage to the ripeness stage as the test material used in the experiment. But further research is still required to clarify why there was higher LA percent compared to CTRL under MAX at F3 than that of F1. Water-soluble carbohydrate (WSC) content along with the activity of epiphytic LAB determines the rate of decline in pH during the early stages of ensiling, which is important for producing stable silage [16]. Woolford [17] concluded that an initial WSC content between 60 and 80 g/kg DM was adequate to produce good-quality grass silage. In this study, WSC in the whole plant corn was 91.2 g/kg DM, which is adequate for producing good-quality silage. All silages had pH values below the threshold of 4, and very little butyric acid was detected in the samples (Table 2). Control silage had the greatest (p<0.05) pH value (3.80), and MAX silage had the lowest value (p<0.05; 3.48), which reflected the differences in their respective LA and acetic acid concentrations, especially for filling one time. Nevertheless, all pH values were less than four, which indicated that all the forage was adequately fermented.

The literature review published by Muck and Kung [18] established that silage microbial inoculants can alter different aspects of silage fermentation, such as pH, lactic and acetic acid concentrations, DM losses, and DM and fiber digestibility, but the level of effect is variable across studies. In the present study, compared to CTRL, the fermentation aids MIX and MAX with homofermentative LAB enhanced the LA fermentation whether the samples were filled one time or three times. Corn silage treated with MAX especially showed significantly higher LA content, acetic acid/propionic acid values and lower pH value, acetic acid, propionic acid, butyrate acid, and total VFA compared to MIX and CTRL, which could be attributed to the higher homofermentative LAB in MAX. Wang et al [19] reported that MIX and MAX can reduce ensiling fermentation time and cause the silage to enter LA fermentation in advance by inhibiting negative bacteria activities degrading WSC in the corn plant and reducing the heat produced by aerobic respiration; therefore, the LAB grow rapidly under low-temperature and anaerobic conditions, and sufficient LA production promotes the reduction in pH, which improves the silage quality. That reduced pH is the reason the acetic acid under MAX and MIX was lower compared to that in CTRL. Higher LA indicated that the homofermentative bacteria in MAX and MIX did dominate the epiphytic heterofermentative LAB population estimated in forage [20].

Usually, silage fermentation produces volatile compounds, which will often cause the apparent DM% of silage to decrease compared to fresh material [21]. In present study, the whole plant corn silage treated with MAX showed the highest DM among all silages. Greater DM maintenance due to applying a microbial additive may be attributed to the higher fermentation efficiency of the homofermentative LAB compared to the effects of epiphytic LAB on forage without an additive. These inoculated bacteria transform sugars into LA without producing secondary metabolites or gases [4]. MAX had some effect on the fiber fraction, which resulted in lower CF, NDF, ADF, and HC, although there were no significant differences compared to CTRL and MIX (Table 3), which probably occurred because of acid hydrolysis of HC due to the reduction in pH from fermentation by LAB [4]. MAX with homofermentative LAB increased residual NFE contents compared to CTRL and MIX, which likely occurred because DM loss by microorganisms was inhibited by MAX, acting as a silage additive to inhibit the use of WSC by undesirable bacteria and reduce silage losses during the early stages of ensiling. These conditions led to the availability of more fermentation substrate for LAB and better fermentation quality [22].

In our study, although no significant differences in DMD, ADFD, and NDFD with the exception of CPD among treatments were observed, MAX slightly increased DMD, ADFD, and NDFD (Table 3) and therefore obtained the higher dDM and dNDF under two filling times (p>0.05) (Table 4). Weinberg et al [23] compared the effects of 10 sources of LAB on *in vitro* DMD and NDFD of corn silages and found that DMD improved with some inoculants, whereas NDFD did not. The authors concluded that this effect might be due to some solubilization of HC during ensiling, which improved DMD but did not change or even decreased the digestibility of the residual NDF. The present study had lower CPD after treatment with MAX, which likely contributed to the acid produced by LAB fermentation reducing the apparent digestibility of CP in the rumen and increasing the proportion of bypass protein flowing to the duodenum. This result is consistent with the data of Broderick [24], who reported that the addition of acid to silages prevents CP hydrolysis and conversion of CP to ammonia.

### Fermentation profile, silage quality and digestible nutrient yield

Knowledge about the effects of ensiling conditions, e.g., delayed sealing and air infiltration during the ensiling process, is still rare [19]. In farming practice, delayed sealing is often necessary as a result of weather conditions making it difficult to collect forage, because there is insufficient forage to fill the silo at one time, and for other reasons, all of which will lead to air infiltration and oxygen residual during the ensilage process, especially if the silo must be filled several times. Residual oxygen in silage can impair the growth rate of some homofermentative bacteria [25], which results in a decline in the LA concentration [26]. Conflicting results have been reported on the effects of delayed sealing and air infiltration on acetic acid and the production of volatile organic compounds, especially ethanol [27]. In our study, silage filled one time was clearly superior with respect to higher LA synthesis, acetic to propionic acid ratios, and lower pH, acetic acid, propionic acid and butyrate acid compared to silage filled three times (Table 2), which contained residual oxygen. This effect may be attributed to prolonged respiration processes by plant enzymes or several epiphytic aerobic microorganisms competing with LAB for fermentable carbohydrates [28]. In addition, the increase in acetic acid observed in silages filled three times (p<0.01) is attributable to the presence of residual oxygen, alcohol and aldehyde dehydrogenases produced by Acetobacter and Gluconobacter, which can convert ethanol produced after ensilage for 60 d to acetic acid [29]. McEniry et al [30] found slightly lower stability and a faster rise in temperature, but these factors were not analyzed in our studies.

Additionally, compared to silage filling times, silage filled one time had higher DM and lower DMLR, EE and Ash (p< 0.01). Although other nutrient indexes also have consistent results, they showed no biological significance (Table 3). Therefore, higher nutrient digestibility (DMD, NDFD, ADFD, and CPD) (Table 3) and digestible nutrient yield (dDM and dNDF) (Table 4) were attained. This finding cannot be discussed in relation to other published data because, to the best of our knowledge, this study is the first time that this factor has been tested in this manner. Thus, delayed sealing may have directly or indirectly affected the metabolic activity of potential n-propanol producers [27]. Unfortunately, additional studies on the effects of residual oxygen or air infiltration on the fermentation process are not available. Finally, large variations in the fermentation pattern, nutrient value, and nutrient digestibility may be responsible for the observed differences among studies investigating the complexity of the silage fermentation process and the inhabitant microbial ecosystem.

## CONCLUSION

After 60 d of ensiling, due to sufficient fermentable substrate and epiphytic LAB in whole plant corn, all corn silages showed good fermentation attributes (pH<4.0). MAX with homofermentative LAB enhanced the LA fermentation, silage quality and nutrient digestibility, compared to CTRL and MIX, and the digestible nutrient yield was also improved, which showed that MAX can be popularized and used in farm corn silage practice in northern China. Silages treated with all additives filled one time, compared to samples filled three times, attained good fermentation qualities; this finding indicates that residual oxygen during ensiling can impair the growth rate of some homofermentative bacteria and lead to a decline in LA concentration and silage quality. Therefore, filling the silo one time is recommended in ensiling process if the conditions are suitable.

## Figures and Tables

**Table 1:** Ingredient composition and nutrient concentrations of the experimental diet

Items	
Ingredient (% As is)	
Corn straw silage	72.96
Corn	14.41
Bran	4.00
Rapeseed meal	3.59
Cottonseed meal	3.86
1% Premix^1)^	0.29
Calcium bicarbonate	0.31
Mineral meal	0.31
Salt	0.28
Total	100.00
Nutrient (% of DM)	
DM (%)	36.2
DE (MJ/kg)	14.1
ME (MJ/kg)	11.5
CP (%)	14.7
Ca (%)	0.76
P (%)	0.65

DM, dry matter; DE, digestible energy; ME, metabolizable energy; CP, crude protein.

1) 1% premix contained mineral elements (mg/kg): Fe (FeSO_4_·7H_2_O), 25; Zn (ZnSO_4_·7H_2_O), 40; Cu (CuSO_4_·5H_2_O), 8; I (KI), 0.3; Mn (MnSO_4_·5H_2_O), 40; Se (Na_2_SeO_3_), 0.2; Co (CoCl_3_·6H_2_O), 0.1; vitamin (IU/kg): vitamin A, 940; vitamin E, 20.

**Table 2:** Effects of filling method and additives on fermentation characteristics (% of dry matter unless noted otherwise) of whole plant corn silage

Measurements	F_1_^1)^			F_3_^1)^			SE	p-value^2)^		
		
	CTRL	MAX	MIX	CTRL	MAX	MIX		FM	ADD	FM×ADD
pH	3.80^a^	3.58^b^	3.48^b^	3.23^c^	3.23^c^	3.23^c^	0.057	<0.001	0.026	<0.001
Latic acid (%)	6.08^e^	8.01^a^	7.96^c^	6.05^f^	7.98^b^	7.63^d^	0.0058	<0.001	<0.001	<0.001
Acetic acid (%)	2.65^b^	1.37^f^	1.89^d^	2.83^a^	1.75^e^	2.01^c^	0.0058	<0.001	<0.001	<0.001
Propionic acid (%)	0.08^b^	0.01^e^	0.03^d^	0.10^a^	0.04^d^	0.06^c^	0.0053	<0.001	<0.001	<0.001
Butyrate acid (%)	0.06^a^	0.01^c^	0.02^c^	0.07^a^	0.04^b^	0.06^a^	0.0053	<0.001	<0.001	<0.001
Volatile fatty acids (%)	2.79^b^	1.39^f^	1.94^d^	3.00^a^	1.83^e^	2.13^c^	0.011	<0.001	<0.001	<0.001
Acetic acid/propionic acid	33.13^c^	137.00^a^	63.00^b^	28.30^c^	43.75^c^	33.50^c^	6.53	<0.001	<0.001	<0.001

SE, standard error.

1) F1, silage filled 1 time; F3, silage filled 3 times; CTRL, no treatment; MAX, treated with Sila-Max (Ralco. Nutrition Inc., Marshall MN, USA); MIX, treated with Sila-Mix (Ralco. Nutrition Inc., USA).

2) FM, main effect of filling method; ADD, main effect of additive treatment; FM×ADD, interaction of filling method and additive treatment.

a–f Means in rows with unlike superscripts differ, p<0.05.

**Table 3:** Nutrient composition (%) of whole plant corn silage as influenced by filling method and additive after 60 d of ensiling

Nutrients	Before ensiling	F_1_^1)^			F_3_^1)^			SE	p-value^2)^		
		
		CTRL	MAX	MIX	CTRL	MAX	MIX		FM	ADD	FM×ADD
DM (%)	25.39	20.08^ab^	20.93^a^	20.48^a^	17.95^d^	19.30^bc^	19.04^c^	0.26	<0.001	0.013	<0.001
DMLR (%)	-	21.23^c^	18.16^c^	19.87^c^	29.75^a^	24.44^b^	25.40^b^	1.04	<0.001	0.015	<0.001
CP (%)	7.30	6.31	6.79	6.49	6.26	6.69	6.38	0.18	0.558	0.221	0.982
EE (%)	1.92	2.03^a^	1.75^bc^	1.57^c^	2.10^a^	1.94^ab^	1.94^ab^	0.06	<0.001	0.002	<0.001
CF (%)	22.47	26.49^ab^	25.98^bc^	25.62^c^	27.38^a^	25.77^bc^	26.67^ab^	1.05	<0.001	0.817	<0.001
Ash (%)	5.53	5.85^b^	5.83^b^	5.77^b^	6.80^a^	6.15^ab^	6.12^ab^	0.23	<0.001	0.273	<0.001
NFE (%)	57.81	48.58	49.64	49.43	47.60	49.25	48.72	0.92	0.376	0.664	0.948
NDF (%)	47.20	40.33^ab^	39.80^ab^	37.43^b^	42.01^a^	39.43^ab^	38.40^ab^	1.25	<0.001	0.181	<0.001
ADF (%)	29.37	26.46	26.44	26.06	26.63	26.22	26.13	0.88	0.995	0.988	0.974
HC (%)	17.83	13.87	13.36	11.38	15.38	13.21	12.27	1.33	0.503	0.358	0.822

SE, standard error; DM, dry matter; DMLR, dry matter lost rate; CP, crude protein; EE, ether extraction; CF, crude fiber; CA, crude ash; NFE, nitrogen free extract; NDF, neutral detergent fiber; ADF, acid detergent fiber; HC, hemicellulose.

1) F1, silage filled 1 time; F3, silage filled 3 times; CTRL, no treatment; MAX, treated with Sila-Max (Ralco. Nutrition Inc., Marshall MN, USA); MIX, treated with Sila-Mix (Ralco. Nutrition Inc., USA).

2) FM, main effect of filling method; ADD, main effect of additive treatment; FM×ADD, interaction of filling method and additive treatment.

a–f Means in rows with unlike superscripts differ, p<0.05.

**Table 4:** Digestibility of silage nutrients by sheep and yield of digestible nutrients as affected by filling method and additive

Nutrients	Before ensiling	F_1_^1)^			F_3_^1)^			SE	p-value^2)^		
		
		CTRL	MAX	MIX	CTRL	MAX	MIX		FM	ADD	FM×ADD
DMD (%)	55.42	69.59^bc^	71.17^a^	67.88^ab^	65.50^d^	65.07^cd^	55.54^d^	3.95	<0.001	0.098	<0.001
ADFD (%)	30.00	44.29^b^	51.24^a^	45.86^b^	35.56^c^	36.71^c^	36.57^c^	1.72	<0.001	0.118	<0.001
NDFD (%)	20.93	40.77^ab^	46.35^a^	41.12^ab^	33.95^b^	36.18^b^	36.08^b^	2.54	<0.001	0.505	<0.001
CPD (%)	71.00	83.72^bc^	88.49^a^	84.84^ab^	83.73^c^	79.56^bc^	82.52^c^	1.39	<0.001	0.031	<0.001
DM (g/kg)	253.90	200.8^a^	209.3^a^	204.8^a^	179.5^c^	193.0^b^	190.4^b^	0.17	<0.001	0.01	<0.001
dDM (g/kg)	140.71	139.74^b^	148.96^a^	139.02^b^	117.57^c^	125.59^c^	105.75^d^	8.20	<0.001	0.01	<0.001
NDF (g/kg)	472.0	403.3^ab^	398.0^ab^	374.3^b^	420.1^a^	394.3^ab^	384.0^ab^	7.09	<0.001	0.50	<0.001
dNDF (g/kg)	98.79	164.43^ab^	184.47^a^	153.91^ab^	142.62^b^	142.66^b^	138.55^b^	7.62	<0.001	0.34	<0.001

SE, standard error; DMD, dry matter digestibility; ADFD, acid detergent fiber digestibility; NDFD, neutral detergent fiber digestibility; CPD, crude protein digestibility; dDM, yield of digestible dry matter; dNDF, yield of digestible neutral detergent fiber.

1) F1, silage filled 1 time; F3, silage filled 3 times; CTRL, no treatment; MAX, treated with Sila-Max (Ralco. Nutrition Inc., Marshall MN, USA); MIX, treated with Sila-Mix (Ralco. Nutrition Inc., USA).

a–d Means in rows with unlike superscripts differ, p<0.05.
